# Effects of butyric acid and arsenic on isolated pancreatic islets and liver mitochondria of male mouse

**Published:** 2017

**Authors:** Akram Ahangarpour, Ali Akbar Oroojan, Mohsen Rezae, Mohammad Javad Khodayar, Soheila Alboghobeish, Marzieh Zeinvand

**Affiliations:** 1 *Health Research Institute, Diabetes Research Center, Department of Physiology, Ahvaz Jundishapur University of Medical Sciences, Ahvaz, Iran*; 2 *Department of Physiology, Student Research Committee of Ahvaz Jundishapur University of Medical Science, Ahvaz, Iran*; 3 *Department of Toxicology, Faculty of Medical Sciences, Tarbiat Modares University, Tehran, Iran*; 4 *Department of Pharmacology and Toxicology, School of Pharmacy, Jundishapur University of Medical Sciences, Ahvaz, Iran *; 5 *Department of Pharmacology, School of Pharmacy, Student Research Committee of Ahvaz Jundishapur University of Medical Sciences, Ahvaz, Iran *; 6 *Department of Toxicology, School of Pharmacy, Student Research Committee of Ahvaz Jundishapur University of Medical Sciences, Ahvaz, Iran *

**Keywords:** Butyric acid, Arsenic, Liver mitochondria, Islet, Mouse

## Abstract

**Aim:**

The aim of the present study was to evaluate the different doses of Butyric acid (BA) and Arsenic (As) in liver mitochondria oxidative stress and pancreatic islet insulin secretion of male mouse.

**Background:**

BA is found in many foods and As as a toxic metal is present in drinking water. They can induce oxidative stress in tissues.

**Methods:**

In this experimental study, Liver mitochondria were isolated by administration of the different centrifugation method and pancreatic islets were isolated by collagenase method. Mitochondria were incubated by BA (35, 75, 150, 300 μM) and As (20, 50, 100, 200 μM) as the islets were incubated by BA (250, 500, 1000, 1500 μM) and As (50, 100, 200 μM) for 1 hour. At the end of the experiment, mitochondrial viability and membrane potential, ROS, MDA, GSH and islets insulin secretion were measured by their specific methods.

**Results:**

BA and As administration increased mitochondrial levels of ROS, MDA and decreased GSH and pancreatic islet insulin secretion in a dose dependent manner (*p*<0.05). The doses of BA 75μM and As 100μM have been revealed the most mitochondria toxic concentrations. Also, the doses of 1000μM for BA and 100μM for As were considered as reducing concentrations for islets insulin secretion. Additionally, co administration of them intensified more these effects

**Conclusion:**

Alone or in combination administration of BA and As induced oxidative stress in liver mitochondria and decreased insulin secretion of pancreatic islets.

## Introduction

Butyric acid (BA) as an anaerobic fermentation product has been found in milk, butter, parmesan cheese. Milk fatty acid contents such as BA immediately absorbed in the upper intestine and taken up by the liver immediately. The effective concentration of BA depends on the rate of hydrolysis, volume of fluid transit, and the balance of material in the meal. So, it is highly possible that several tissues are exposed to millimolar levels of BA followed by administration of milk fat (1). As the majority of BA remains in the liver, one clinical study revealed that physiological concentrations of short chain fatty acid have been recorded about 378±70µM in the portal blood of sudden death victims (2). Previous study indicated that daily administration of BA induced hepatotoxicity in the bulls through the increase hepatic enzymes levels such as aspartate aminotransferase, alanine aminotransferase and gamma-glutamyltransferase (3). Further, this fatty acid is an extracellular metabolite that is commonly found in the mouth, vagina and gut. BA produced through the butyrate kinase or butyryl-CoA: acetate CoA-transferase pathways and its retention indicated an alteration in calcium homeostasis and induce oxidative stress in the jugular blood mitochondria (4, 5). There is no scientific evidence about this fatty acid effect on liver mitochondrial oxidative stress (5). One study revealed that BA couldn’t induce any changes in islet insulin secretion at a dose of 10µM. Hence, more study is required to clarify the effective and exact doses of this fatty acid on insulin secretion (6).

Arsenic (As) is an Earth’s crust toxic metal and distributed in the soil, drinking water and food (7). Between the two forms of organic and inorganic arsenic, the inorganic is more toxicant (8). Arsenic exposure has been shown to be associated with several diseases such as diabetes mellitus, arteriosclerosis, hepatomegaly, peripheral vascular disease, cardiovascular diseases, hypertension, and numerous cancers (9-12). Previous study indicated that the liver is the main target of arsenic toxicity (13). In addition, some evidence showed that the mitochondria are the target organelle for metal toxicity (14-16). Further, arsenic increased ROS formation, glutathione oxidation and decreased mitochondrial membrane potential in liver (17). The production of the superoxide anion and ROS is one of the mechanisms of arsenic mitochondrial toxicity due to its mobility (18).

In our previous study it was revealed that concomitant administration of As and high fat diet (HFD) induced a different form of type2 diabetes that characterized by deficiencies of oral glucose tolerance test (OGTT) and islet insulin secretion through the mitochondrial oxidative stress enhancement (19). So, since drinking water contains As in various countries and BA belongs to the HFD or several foodstuffs, and there is no scientific study about the exact dose of these metal and fatty acid on liver mitochondrial oxidative stress or islets insulin secretion, present study conducted to evaluate the effects of different concentrations of BA and As on liver mitochondria and pancreatic islets to determine the exact doses that able to induce mitochondrial oxidative stress or reduce islets insulin secretion.

## Materials and Methods

**Study design**

*Experimental Animals*

Adult male NMRI mice (30-35 g) were obtained from the animal facility of the Ahvaz Jundishapur University of Medical Science (AJUMS), which is completelyattributed by AJUMS animal care guidelines with an ethics committee grantee No. IR.AJUMS.REC.1394.604.Theyhoused in an air-conditioned room with controlled temperature of 20±4°C, humidity of 70-80% and maintained on a 12:12 h light cycle with free access to food and water. 

**Chemicals**

Sodium arsenite (NaAsO2),Butyric acid(short chain fatty acid), 4-2-hydroxyethyl-1-piperazineethanesulfonic acid (HEPES), DMSO, D-mannitol, thiobarbutiric acid (TBA), MTT (3-[4,5-dimethylthiazol-2-yl]-2,5-diphenyltetrazolium bromide), dithiobis-2-nitrobenzoic acid (DTNB), reduced glutathione (GSH), 2′,7′-dichlorofluorescein diacetate (DCFH-DA), sodium succinate, sulfuric acid, Tetramethoxypropane (TEP), sucrose, KCl, Na2HPO4, MgCl2, MnCl2,potassium phosphate,Rhodamine 123 (Rh 123), Coomassie blue, ethylenediaminetetraacetic acid(EDTA) and bovine serum albumin (BSA) were purchased from SigmaChemical Co. (St. Louis, MO, USA). 

**Experimental procedure**

*Mitochondrial isolation*

Liver mitochondrial isolation was performed using centrifugation method as described previously (20). The animals were sacrificed by decapitation and their liver were rapidly removed, washed with cold buffer and cut into small pieces. Liver pieces were homogenized in an ice-cold isolation buffer containing sucrose 70 mM, mannitol 200 mM, HEPES 10 mM, EGTA 1 mM, and BSA 0.1% (pH ¼ 7.4) (21). The nuclei and broken cell debris where sediment by centrifuging at 1500×g for 10 min at 4ºC and the pellet was discarded. Then, the supernatant centrifuged at 10,000×g for 10 min and the superior layer was carefully discarded. The mitochondrial pellets were washed by suspending in the isolation buffer and centrifuged again at 10,000×g for 10min. Ultimately, these mitochondria pellets were suspended in Tris buffer containing (Tris-HCl 0.05 M, sucrose 0.25 M, KCl 20 mM, MgCl2 2.0 mM, and Na2HPO4 1.0 mM, pH 7.4) at 4°C. Protein concentrations were determined according to the Bradford and Coomassie blue protein binding methods by using BSA as the standard sample (22). Mitochondria were prepared fresh for each test and used in 4 h. In order to prepare highest quality of mitochondrial isolation, all steps have been done on ice. The concentrations of BA (0 (control group), 35, 75, 150, 300 μM) and As (0 (control group), 20, 50, 100, 200 μM) were chosen based on the previous study (23-26). The mitochondrial fractions were incubated in Tris buffer with different doses of As and BA for 1 h (21).

*Islet isolation*

Pancreatic islets were isolated from the animals by the collagenase digestion method (27). Pancreas tissue was removed and transferred into a petri dish containing 5mL Krebs-bicarbonate buffer (NaCl 115mM, KCl 5mM, CaCl_2_2.56mM, MgCl_2_1mM, NaHCO_3_10mM, HEPES 15mM, supplemented with 0.5% bovine serum albumin (BSA) and stabled with a mixture of 95% oxygen, 5% carbon dioxide, pH 7.4) and centrifuged at 100×g for 5 min (28). For the sake of isolated islet purification from exocrine tissues, collagenase type P (Roch Company, Germany) (1-2 mg/pancreas) was added to the solution and put in a shaking water bath 800 oscillations shake for 5-10 min at 37°C. Then, 15mL of cold Krebs-bicarbonate buffer was added to stop collagenase digestion and centrifuged at 500×g for 5 min. The supernatant of the sample was removed to a blackened petri dish, and islets dissection was carried out manually under stereomicroscope observation using drawn-out glass pipette (29). 

**Experimental outcomes**

*Mitochondrial Viability Assessment*

The mitochondrial total dehydrogenase activity)complex II( was assayed by measuring the reduction of MTT (3-[4,5-dimethylthiazol-2-yl]-2,5-diphenyltetrazoliumbromide) to formazan. At the first, 100 μL of mitochondrial suspension (0.5 mg protein/mL) was incubated with different concentrations of As or BA for 1 hour. Then, 50 μL MTT 0.4% was added to this medium and incubated at 37°Cfor 30 min. Finally, the product of purple formazan crystals was dissolvedin100 μL dimethyl sulfoxide (DMSO), and the absorbance was measured at 570 nm by a spectrophotometer (UV-1650PCShimadzu) (30, 31).

*Mitochondrial Membrane Potential Assessment*

Mitochondrial membrane potential (MMP) was measured by mitochondrial uptake of rhodamine 123 with fluorescence spectrophotometer. The mitochondrial fractions (0.5 mg protein/mL) were incubated with various concentrations of As and BA for 1 h. Then, 10 μM of rhodamine 123 (Rh123) was added to the mitochondrial solution. The capacity of mitochondria for Rh123 uptake the was calculated as a difference between control and treated mitochondria by the measurement of Fluorescence intensity spectrofluorometerically (LS50B PerkinElmer, Waltham, Massachusetts, USA; the excitation and emission wavelength of 490 nm and 535 nm) (32, 33).

*Mitochondrial ROS Assessment*

The levels of ROS were measured by adding 1 mL of dichloro-dihydro-fluorescein diacetate (DCFH-DA) 3.32M to the 1 mL of isolated mitochondria sample. DCFH-DA enters into mitochondria and hydrolyzes to non fluorescent DCFH. Then, oxidized to form highly fluorescent 2,7-dichlorofluorescein through the reaction with ROS. Further, the fluorescence intensity was measured by a fluorescence spectrophotometer (UV-1650PC SHIMADZU, Kyoto, Japan; Ex ¼ 500 nm, Em ¼ 520 nm) (34).

*Lipid Peroxidation Measurement*

The content of MDA was determined using thiobarbituric acid reactive substances (TBARS) formation. The mitochondrial fractions (0.5 mg protein/mL) were incubated with various concentrations of As and BA for 1 h at 30 °C. Then, 1mL of the separated mitochondrial solution was mixed with 250µL trichloroacetic acid (70%) and centrifuged at 3000×g for 15 min. The supernatant was added to 1mL TBA (0.8%) and placed in a boiling water bath for 30 min. The sample absorbance was read at 412 nm on a spectrophotometer. Values were expressed as µg/mg protein. Since 99% of the TBARS were malondialdehyde (MDA), TBARS concentrations of the samples were calculated from a standard curve using 1, 1, 3,3-tetramethoxypropane (35,36).

*Mitochondrial GSH Measurement*

Reduced glutathione (GSH) was analyzed using DTNB (5, 5'-dithiobis-2-nitrobenzoic acid) as an indicator. The mitochondrial sample (0.5 mg protein/mL) was incubated with various concentrations of As and BA for 1 h at 30°C. Then, 0.1 mL of mitochondrial fractions was added into the 0.1 mol/L phosphate buffers with 0.04% DTNB in a total volume of 3.0 mL (pH 7.4). The developed yellow color was spectrophotometrically read at 412 nm (UV-1601PC, Shimadzu, Japan) (32).

*Islet’s insulin secretion*

Ten manually dissected islets were transferred into the 2mL micro tubes containing Krebs-bicarbonate buffer with 5.6mM of d-Glucose concentrations (similar to fasting blood glucose)(37).The different concentrations of BA (0 (control group), 250, 500, 1000, 1500 μM) and As (0 (control group), 50, 100, 200 μM) were added to the isletsmedium and incubated at 37°C for 90 min. After incubation the samples centrifuged at 100×g for 5 min and, 0.9 mL of sample’s supernatant maintained at −70°C until the insulin secretion assay was performed. Each micro tube contains 10 islets and this in vitro protocol repeated 8 times for each BA and As concentrations (38). Insulin secretion was measured using enzyme-linked immunosorbent assay (ELISA) method with its commercial assay kit (Linco Research, St. Charles, MO).

**Ethical statement**

Prior to the experiment, all protocols were confirmed to be in accordance with the Guidelines of Animal Ethics Committee of Ahvaz Jundishapur University of Medical Sciences.This research was approved by the ethical committee of the Ahvaz Jundishapur University of Medical Sciences with an ethics committee number (IR.AJUMS.REC.1394.604).

**Statistical Assessment**

The obtained data were statistically analyzed by using SPSS software as mean ± standard error of mean (SEM) with one-way analysis of variance (ANOVA) and, post hoc least significant difference (LSD) tests. Further, the differences were considered statistically significant at *p*<0.05.

## Results

**Effects of BA** **and** **As** **exposure** **on liver mitochondria viability**

**Figure 1:**
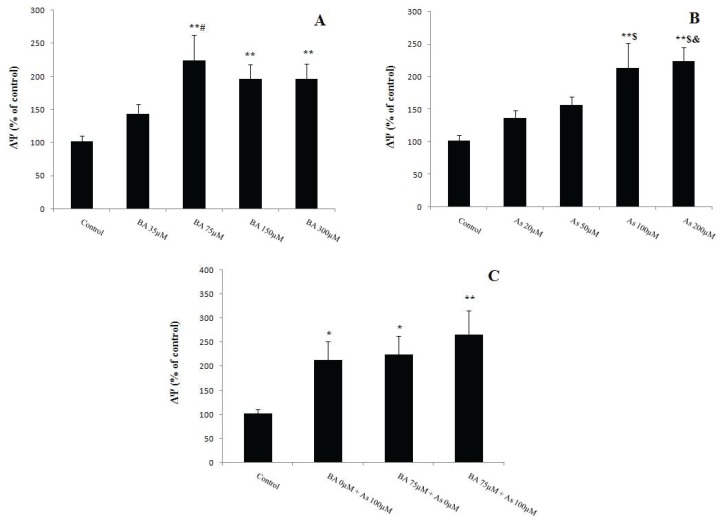
Effects of Butyric acid and Arsenic on mitochondrial viability of the liver (MTT). (A) Effects of BAon MTT,(B) Effects of As on MTT, (C) Effects of BA+As on MTT. Data are expressed as the mean ± SEM for 12 mice in each group. *: compared to control, #: compared to BA 35 µM. # and *p<0.05,**p<0.01, *** *p*<0.001. BA; Butyric acid, As; Arsenic

MTT, as mitochondrial viability variable, was assessed by the activity of succinate dehydrogenase (complex II). The results of BA administration showed a significant decrease in the MTT level at a dose of 35µM (*p*<0.05), 75µM (*p*<0.001), 150µM (*p*<0.01) and 300µM (*p*<0.001) compared to BA 0µM. Further, BA 300 µM utilization reduced this variable when compared to BA 35 µM (*p*<0.05) (Figure 1A). Also, the levels of MTT decreased in As 50µM (*p*<0.05) and 100, 200µM (*p*<0.01) in comparison with As 0µM (Figure 1B). Further, the results of BA 75µM and As 100 µM concomitant administration revealed similar effect on liver’s mitochondrial MTT levels when compared to BA 0µM + As 0µM (*p*<0.001) (Figure 1C).

**Effect of BA** **and** **As** **exposure** **on mitochondrial membrane damage**

**Figure 2:**
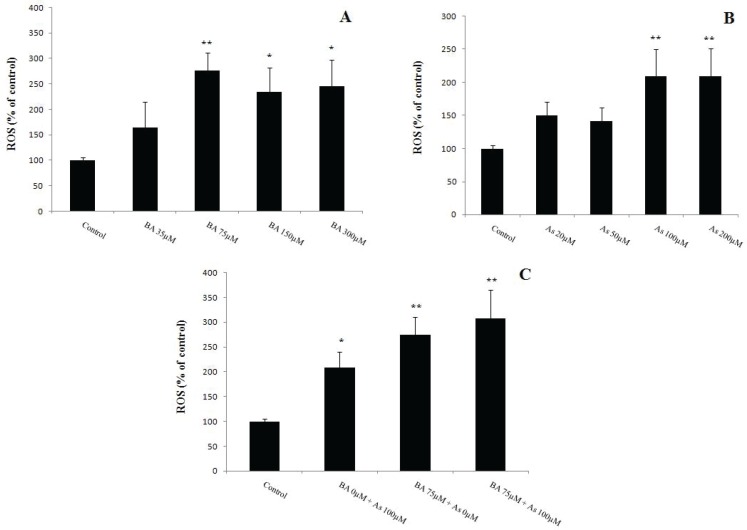
Effects of Butyric acid and Arsenic on mitochondrial membrane potential collapse of the liver (ΔΨ). (A) Effects of BA on ΔΨ, (B) Effects of As on ΔΨ, (C) Effects of BA+As on ΔΨ. Data are expressed as the mean ± SEM for 12 mice in each group.*: compared to control, #: compared to BA 35 µM, $: compared to As 20 µM, &: compared to As 50 µM. #, $, & and * p<0.05,** p<0.01.BA; Butyric acid, As; Arsenic

MMP is a variation of the mitochondrial membrane damage which known as ΔΨ. So, present data indicated that the BA 75, 150, 300µM and As 100, 200µM administration induced a significant increase in ΔΨ levels when compared to BA 0µM and As 0µM respectively (*p*<0.01). This variable increased in BA 75 µM group compared to BA 35 µM (*p*<0.05). Also, As 100, 200 µM administration increased the levels of ΔΨ in comparison with As 20, 50 µM (*p*<0.05) (Figures 2A and 2B). Finally, together mitochondrial incubation by BA 75µM and As 100µM for 1 hour (21) revealed similar effect on the enhancement of its membrane damage compared to BA 0µM + As 0µM (*p*<0.01) (Figure2C). 

**Effect of BA** **and** **As** **exposure** **on mitochondrial oxidative stress**

**Figure 3:**
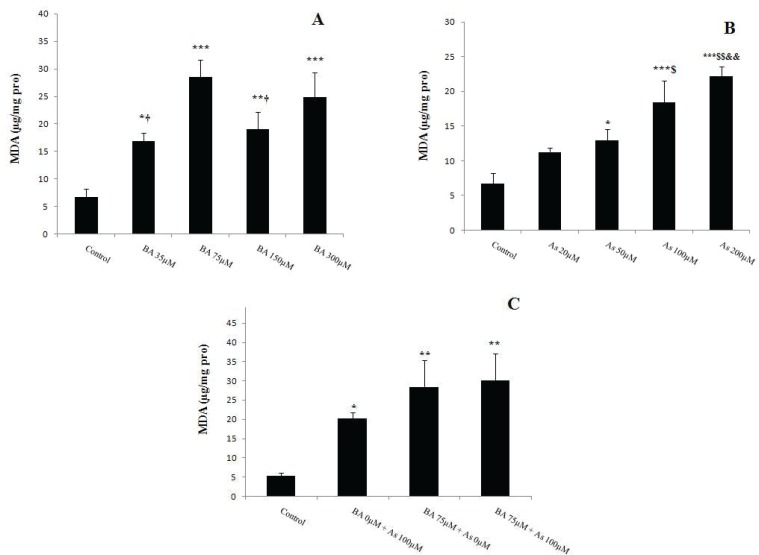
Effects of Butyric acid and Arsenic on mitochondrial ROS formation in the liver. (A) Effects of BA on ROS, (B) Effects of As on ROS, (C) Effects of BA+As on ROS. Data are expressed as the mean ± SEM for 12 mice in each group. *: compared to control, * p<0.05,** p<0.01.BA; Butyric acid, As; Arsenic

Oxidative stress was assessed by measurement of the ROS throughout DCFH-DA oxidation, and the results indicated that 1 hour incubation of BA 75µM (*p*<0.01), 150 and 300µM (*p*<0.05) increased ROS levels in comparison with BA 0µM (Figure 3A). Further, As administration at doses of 100 and 200µM revealed the same effects when compared to As 0µM (*p*<0.01) (Figure 3B). Moreover, concomitant incubation of BA 75µM and As 100µM showed a tendency to more increase in ROS levels compared to BA 0µM + As 0µM (*p*<0.01) (Figure3C).

**Effect of BA** **and** **As** **exposure** **on mitochondrial MDA level**

**Figure 4:**
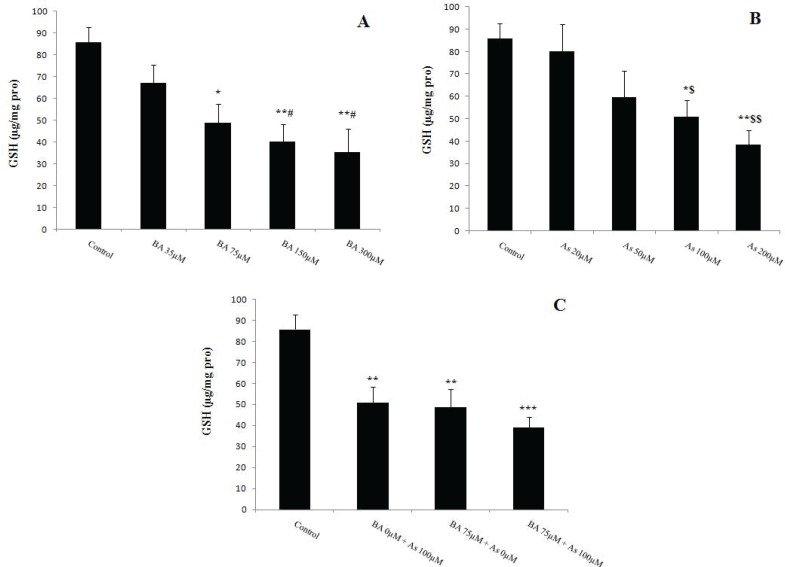
Effects of Butyric acid and Arsenic on mitochondrial MDA level of the liver. (A) Effects of BA on MDA, (B) Effects of As on MDA, (C) Effects of BA+As on MDA. Data are expressed as the mean ± SEM for 12 mice in each group.*: compared to control, †: compared to BA 75µM, $: compared to As 20 µM, &: compared to As 50 µM. †, $ and * p<0.05,$$, && and** p<0.01, *** p<0.001.BA; Butyric acid, As; Arsenic

**Figure 5:**
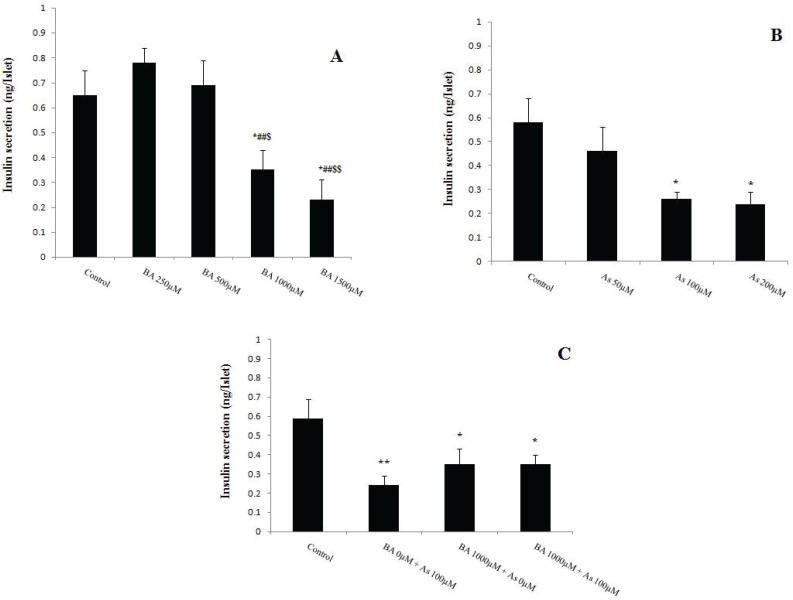
Effects of Butyric acid and Arsenic on mitochondrial GSH level of the liver. (A) Effects of BA on GSH, (B) Effects of As on GSH, (C) Effects of BA+As on GSH. Data are expressed as the mean ± SEM for 12 mice in each group. *: compared to control, #: compared to BA 35 µM, $: compared to As 20 µM. #, $ and * p<0.05,$$ and ** p<0.01, *** p<0.001.BA; Butyric acid, As; Arsenic

MDA levels increased in BA 35µM (*p*<0.05), 75µM (*p*<0.001), 150µM (*p*<0.01) and 300µM (*p*<0.001) when compared to BA 0µM. Also, the same effect was observed after BA 75 µM administration compared to BA 35, 150 µM (*p*<0.05) (Figure 4A). This peroxidation levels increased after 1 hour (21) mitochondrial incubation with As 50µM (*p*<0.05), 100 and 200µM (*p*<0.001) compared to As 0µM,. Further, MDA showed a significant increase in As 20, 50 µM group compared to As 200 µM (*p*<0.01) (Figure 4B) and this variable became more evident after concomitant administration of BA 75µM and 100µM (*p*<0.01) compared to BA 0µM + As 0µM (Figure 5C).

**Effect of BA** **and** **As** **exposure** **on mitochondrial GSH level**

Glutathione is an important antioxidant that measured by DTNB as an indicator. Present results showed a significant decrease in mitochondrial GSH levels in BA 75µM (*p*<0.05), 150 and 300µM (*p*<0.01) versus to BA 0µM. This antioxidant decreased in BA 150, 300 µM when compared to BA 35 µM (*p*<0.05) (Figure 5A). Further, the same effect was observed in As 100µM group when compared to As 0 and 20µM (*p*<0.05) and 200µM (*p*<0.01) incubation (Figure 5B). Moreover, concomitant administration of BA 75µM and As 100µM decreased more mitochondrial GSH level (*p*<0.001) (Figure5C). 

**Figure 6:**
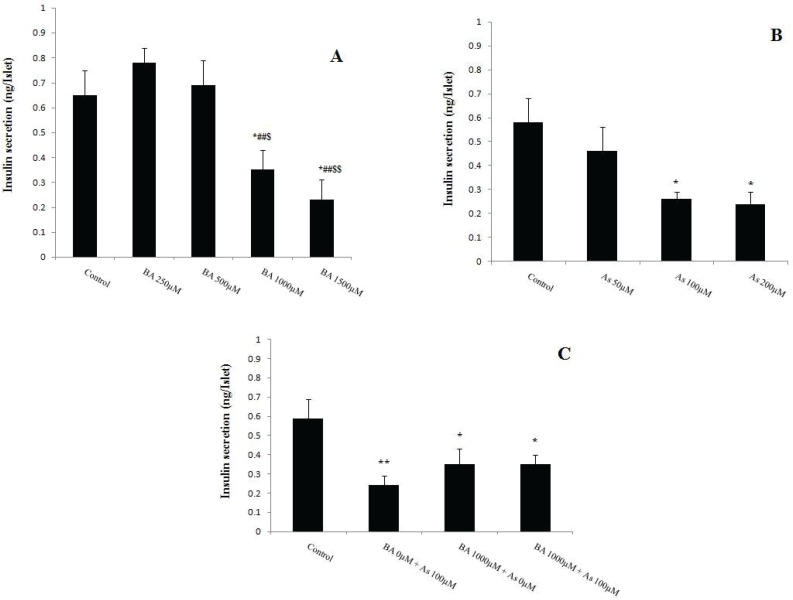
Effects of Butyric acid and Arsenic on insulin secretion of pancreatic islets. (A) Effects of BA on insulin secretion, (B) Effects of As on insulin secretion, (C) Effects of BA+As on insulin secretion. Data are expressed as the mean ± SEM for 12 mice in each group. *: compared to control,#: compared to BA 250 µM, $: compared to BA 500 µM. $ and * p<0.05, ##, $$ and ** p<0.01.BA; Butyric acid, As; Arsenic

**Effect of BA** **and** **As** **exposure** **on** **islets insulin secretion**

Pancreatic islet insulin secretion, known as beta cell function, was measured after 90 min incubation with BA and As. The results revealed that the BA 1000, 1500µM and As 100, 200µM decreased this variable in comparison with BA 0µM and As 0µM respectively (*p*<0.05). Also, BA 1000, 1500 µM administration showed a significant decreased in insulin secretion of pancreatic islets when compared to BA 250 µM (*p*<0.01) and 500 µM (*p*<0.05, *p*<0.01 respectively) (Figure 6A and 6B). Moreover, co administration of BA 1000µM and As 100µM showed similar effects on this secretion when compared to BA 0µM + As 0µM (*p*<0.05) (Figure 6C).

## Discussion

Present data indicated that alone BA and As administration induced liver mitochondrial oxidative stress via increase MDA, ROS, and decrease GSH levels in a concentration dependent manner. Further, alone administration of these agents reduced pancreatic islet insulin secretion dose dependently. Dose responses of BA and As revealed that the maximum mitochondrial oxidative stress-induced concentrations are 75µM for BA and 100µM for As. Moreover, the maximum doses of these agents that able to reduce islet insulin secretion were 1000µM for BA and 100µM for As. However, the results of BA and As co 

administration indicated that the combination of them produced a more tendency to increase mitochondrial oxidative stress and decrease pancreatic islet insulin secretion, but, these effects were not statistically significant in alone administration of them.

The hepatic cell mitochondrial is exposing to ROS damage via fatty acid oxidation. Serum MDA level increased through the severity of liver disease (39) and Glutathione is the first line of antioxidant defense against As damage (40). In accordance with present study, Zgorzynska, *et al.* showed that BA induced oxidative stress via over generation of ROS, decreased cell viability and mitochondrial membrane potential in primary human fibroblasts of gingival tissue(41).However, in vivo study of Cummings, *et al.* revealed a 375µM concentration of BA in the liver of sudden death victims(2), but present in vitro study showed that this fatty acid has an oxidative stress effects at a dose of 75µM. So, since BA is found in milk, butter and parmesan cheese, the administration of these foods should be more attention, however, further investigations are required to elucidate the specific absorbed concentration of BA in the liver after administering a meal of mentioned foods.

Several studies have been demonstrated that As exposure could induce severe toxicity in the liver by generating free radicals. Also, during As administration oxidative stress-mediated mitochondrial damage has been occurring (42). So, the present study showed that acute liver incubation of As can induce similar effects on the mitochondrial destruction like chronic in vivo administration. Hence, consists with our study Dutta, *et al.* showed that As utilization induced liver tissue damage through the increase oxidative stress, lipid peroxidation and decrease GSH levels. So, it could be suggested that animals treated by As aggravate liver mitochondrial damage and viability through the oxidative stress and reduce the GSH level (40).

Increased metabolism of free fatty acids through the mitochondrial oxidation can cause to mitochondrial damage. It is certainly revealed that the islets are more susceptible to lipotoxic damage at normal glucose levels (43). Low sub toxic concentrations of As and its methylated trivalent metabolites may induce adverse effects on pancreatic beta cells and inhibit glucose stimulated insulin secretion (44). Also, one of the subjects to injury from oxidative stress is beta cells insulin secreting. ROS formation is implicated in islet dysfunction or death caused by autoimmune attack and the actions of cytokines. Since beta cells have a limited defense against excess ROS production, they are more susceptible to the oxidative damages compared with other cell types (45).

However, Itoh, *et al.* study demonstrated that the islet insulin secretion did not change at a dose of BA 10µM (6), but present research revealed the exact toxic dose of BA on this secretion. Further, it could be suggested that this fatty acid and As administration may reduce insulin secretion through the imbalance between redox system and lipotoxicity. Therefore, BA and As have similar effects on induction of liver’s mitochondrial oxidative stress and reduction of islet insulin secretion. Hence, it could be suggested that co-administration of them intensified this situation of mitochondria.

The main limitations in this work are the liver crucial homogenization step and protocols, because less force is required compared to hard tissues such as muscle. Also, since many liver samples were prepared simultaneously, the centrifugation steps of them become difficult and time-consuming. Moreover, the loss of some healthy islets has been occurred through the inevitable time consuming of islet isolation procedure.

In conclusion, present data established the highest in vitro dose of BA and As that decreased islets insulin secretion and induced mitochondrial oxidative stress in liver through the imbalance between oxidation factors formation and antioxidant defenses. So, according to the presence of BA in dairy food such as milk, butter and parmesan cheese, the consumption of them should be accompanied with more caution. Finally, since specific tissue oxidative damage may be occurred through the As administration, further studies are required to remove this metal from drinking water. 

## References

[1] Smith JG, Yokoyama WH, German JB (1998). Butyric acid from the diet: actions at the level of gene expression. Crit Rev Food Sci Nutr.

[2] Cummings JH, Pomare EW, Branch WJ, Naylor CP, Macfarlane GT (1987). Short chain fatty acids in human large intestine, portal, hepatic and venous blood. Gut.

[3] Dabbagh MN, Fürll M, Schäfer M (1989). Subacute butyric acid burden in cattle 1 Clinical results and effects on the carbohydrate-fat metabolism and the liver function of young fattening bulls [in German]. Arch Exp Veterinar Med.

[4] Cueno ME, Imai K, Tamura M, Ochiai K (2014). Butyric acid-induced rat jugular blood cytosolic oxidative stress is associated with SIRT1 decrease. Cell Stress Chaperones.

[5] Cueno ME, Kamio N, Seki K, Kurita-Ochiai T, Ochiai K (2015). High butyric acid amounts induce oxidative stress, alter calcium homeostasis, and cause neurite retraction in nerve growth factor-treated PC12 cells. Cell Stress Chaperones.

[6] Itoh Y, Kawamata Y, Harada M, Kobayashi M, Fujii R, Fukusumi S (2003). Free fatty acids regulate insulin secretion from pancreatic beta cells through GPR40. Nature.

[7] Schönfeld P, Wojtczak L (2007). Fatty acids decrease mitochondrial generation of reactive oxygen species at the reverse electron transport but increase it at the forward transport. Biochimica et Biophysica Acta.

[8] Lesnefsky EJ, Moghaddas S, Tandler B, Kerner J, Hoppel CL (2001). Mitochondrial dysfunction in cardiac disease: ischemia–reperfusion, aging, and heart failure. J Mol Cell Cardiol.

[9] Mathews V, Binu P, Sauganth Paul M, Abhilash M, Manju A, Harikumaran Nair R (2012). Hepatoprotective efficacy of curcumin against arsenic trioxide toxicity. Asian Pac J Trop Biomed.

[10] Dwivedi N, Mehta A, Yadav A, Binukumar BK, Gill KD, Flora SJ (2011). MiADMSA reverses impaired mitochondrial energy metabolism and neuronal apoptotic cell death after arsenic exposure in rats. Toxicol Appl Pharmacol.

[11] Ramanathan K, Shila S, Kumaran S, Panneerselvam C (2003). Ascorbic acid and a-tocopherol as potent modulators on arsenic induced toxicity in mitochondria. J Nutrit Biochem.

[12] Niedzwiecki MM, Hall MN, Liu X, Slavkovich V, Ilievski V, Levy D (2014). Interaction of plasma glutathione redox and folate deficiency on arsenic methylation capacity in Bangladeshi adults. Free Rad Biol Med.

[13] Yen CC, Ho TJ, Wu CC, Chang CF, Su CC, Chen YW (2011). Inorganic arsenic causes cell apoptosis in mouse cerebrum through an oxidative stress-regulated signaling pathway. Arch Toxicol.

[14] Flora SJ (2011). Arsenic-induced oxidative stress and its reversibility. Free Rad Biol Med.

[15] Ambrosio F, Brown E, Stolz D, Ferrari R, Goodpaster B, Deasy B (2014). Arsenic induces sustained impairment of skeletal muscle and muscle progenitor cell ultra structure and bioenergetics. Free Rad Biol Med.

[16] Liu J, Waalkes MP (2008). Liver is a target of arsenic carcinogenesis. Toxicol Sci.

[17] Belyaeva EA, Korotkov SM, Saris NE (2011). In vitro modulation of heavy metal-induced rat liver mitochondria dysfunction: a comparison of copper and mercury with cadmium. J Trace Elem Med Biol.

[18] Hosseini MJ, Shaki F, Ghazi-Khansari M, Pourahmad J (2013). Toxicity of arsenic (III) on isolated liver mitochondria: a new mechanistic approach. Iran J Pharm Res.

[19] Ahangarpour A, Alboghobeish S, Rezaei M, Khodayar MJ, Oroojan AA, Zaidy Vand M (2016). Evaluation of diabetogenic mechanism of high fat diet in combination with Arsenic exposure in male mice. Iran J Pharm Res.

[20] Bustamante J, Nutt L, Orrenius S, Gogvadze V (2005). Arsenic stimulates release of cytochrome c from isolated mitochondria via induction of mitochondrial permeability transition. Toxicol Appl Pharmacol.

[21] Keshtzar E, Khodayar MJ, Javadipour M, Ghaffari MA, Bolduc DL, Rezaei M (2015). Ellagic acid protects against arsenic toxicity in isolated rat mitochondria possibly through the maintaining of complex II. Hum Exp Toxicol.

[22] Bradford MM (1976). A rapid and sensitive method for the quantitation of microgram quantities of protein utilizing the principle of protein-dye binding. Anal Biochem.

[23] Pourahmad J, Rabiei M, Jokar F, O›brien PJ (2005). A comparison of hepatocyte cytotoxic mechanisms for chromate and arsenite. Toxicology.

[24] Furuno T, Kanno T, Arita K, Asami M, Utsumi T, Doi Y (2001). Roles of long chain fatty acids and carnitine in mitochondrial membrane permeability transition. Biochem Pharmacol.

[25] Penzo D, Tagliapietra C, Colonna R, Petronilli V, Bernardi P (2002). Effects of fatty acids on mitochondria: implications for cell death. Biochimica et Biophysica Acta.

[26] Korge P, Honda HM, Weiss JN (2003). Effects of fatty acids in isolated mitochondria: Implications for ischemic injury and cardio protection. Am J Physiol Heart Circ Physiol.

[27] Sutton R, Peters M, McShane P, Gray DWR, Morris PJ (1986). An improved method for the isolation of islets of Langerhans from the adult rat pancreas. Transplant Proc.

[28] Amaral AG, Rafacho A, Machado de Oliveira CA, Batista TM, Ribeiro RA, Latorraca MQ (2010). Leucine supplementation augments insulin secretion in pancreatic islets of malnourished mice. Pancreas.

[29] O’Dowd JF (2009). The isolation and purification of rodent pancreatic islets of Langerhans. Methods Mol Biol.

[30] Rezaei M, Salimi A, Taghidust M, Naserzadeh P, Goudarzi G, Seydi E (2014). A comparison of toxicity mechanisms of dust storm particles collected in the southwest of Iran on lung and skin using isolated mitochondria. Toxicol Environ Chem.

[31] Shaki F, Hosseini MJ, Ghazi-Khansari M, Pourahmad J (2012). Toxicity of depleted uranium on isolated rat kidney mitochondria. Biochimica et Biophysica Acta.

[32] Naserzadeha P, Hosseini MJ, Arbabia S, Pourahmad J (2015). A comparison of toxicity mechanisms of cigarette smoke on isolated. Mitochondria Obtained from Rat Liver and Skin. Iran J Pharm Res.

[33] Baracca A, Sgarbi G, Solaini G, Lenaz G (2003). Rhodamine 123 as a probe of mitochondrial membrane potential: Evaluation of proton flux through F0 during ATP synthesis. Biochim Biophys Acta.

[34] Hassani S, Yaghoubi H, Khosrokhavar R, Jafarian I, Mashayekhi V, Hosseini MJ (2015). Mechanistic view for toxic effects of arsenic on isolated rat kidney and brain mitochondria. Biologia.

[35] Sadegh C, Schreck RP (2003). The spectroscopic determination of aqueous sulfite using Ellman's reagent. MIT Undergradu Res J.

[36] Satoh K (1978). Serum lipid peroxide in cerebrovascular disorders determined by a new colorimetric method. Clin Chim Acta.

[37] Jijakli H, Zhang Y, Sener A, Malaisse WJ (2006). Tritiated taurine handling by isolated rat pancreatic islets. Endocrine.

[38] Oliveira CA, Paiva ME, Mota CA, Ribeiro C, Leme JA, Luciano E (2010). Exercise at anaerobic threshold intensity and insulin secretion by isolated pancreatic islets of rats. Islets.

[39] Li S, Tan HY, Wang N, Zhang ZJ, Lao L, Wong CW (2015). The Role of Oxidative Stress and Antioxidants in Liver Diseases. Int J Mol Sci.

[40] Dutta M, Ghosh D, Ghosh AK, Bose G, Chattopadhyay A, Rudra S (2014). High fat diet aggravates arsenic induced oxidative stress in rat heart and liver. Food ChemToxicol.

[41] Zgorzynska E, Wierzbicka-Ferszt A, Dziedzic B, Witusik-Perkowska M, Zwolinska A, Janas A (2015). Docosahexaenoic acid attenuates oxidative stress and protects human gingival fibroblasts against cytotoxicity induced by hydrogen peroxide and butyric acid. Arch Oral Biol.

[42] Prakash C, Kumar V (2016). Chronic arsenic exposure-induced oxidative stress is mediated by decreased mitochondrial biogenesis in rat liver. Biol Trace Elem Res.

[43] Prentki M, Nolan CJ (2006). Islet beta cell failure in type2 diabetes. J Clin Invest.

[44] Huang CF, Yang CY, Chan DC, Wang CC, Huang KH, Wu CC (2015). Arsenic exposure and glucose intolerance / insulin resistance in estrogen-deficient female mice. Environ Health Perspect.

[45] Fridlyand LE, Philipson LH (2004). Does the glucose-dependent insulin secretion mechanism itself cause oxidative stress in pancreatic beta-cells?. Diabetes.

